# Median arcuate ligament syndrome: a rare cause of postprandial abdominal pain – a case report with review of literature

**DOI:** 10.1093/jscr/rjaf1002

**Published:** 2025-12-23

**Authors:** Azeem Farooqui, Manzoor Ahmad, Ahmad Sadiq, Wasif Mohd Ali, Shahzada Alam

**Affiliations:** Department of Surgery, JNMCH, AMU, Aligarh 202002, Uttar Pradesh, India; Department of Surgery, JNMCH, AMU, Aligarh 202002, Uttar Pradesh, India; Department of Surgery, JNMCH, AMU, Aligarh 202002, Uttar Pradesh, India; Department of Surgery, JNMCH, AMU, Aligarh 202002, Uttar Pradesh, India; Department of Surgery, JNMCH, AMU, Aligarh 202002, Uttar Pradesh, India

**Keywords:** median arcuate ligament syndrome, celiac artery, chronic postprandial epigastric pain

## Abstract

Median arcuate ligament syndrome (MALS) is a rare vascular compression syndrome caused by external compression of the celiac artery by the median arcuate ligament. We report a case of a young female with chronic postprandial epigastric pain and significant weight loss, diagnosed with MALS by imaging, and successfully treated surgically. We here report a case of 32-years-old patient of MALS and its management.

## Introduction

Median arcuate ligament syndrome (MALS), also known as celiac artery compression syndrome, is a rare condition caused by compression of the celiac trunk by the median arcuate ligament. It typically presents with postprandial abdominal pain, weight loss, and occasionally an abdominal bruit. Due to its vague symptoms, MALS is often misdiagnosed or diagnosed late [1]. It is considered rare, with estimated incidence ranging between 2 per 100 000 and 5 per 100 000 population per year in some studies, but this varies widely across regions and depending on diagnostic methods used [2, 3]. Anatomical compression of the celiac artery by the median arcuate ligament is seen in 10%–24% of the general population on imaging studies (such as computed tomography (CT) or magnetic resonance (MR) angiography) [1, 4]. However, only a small subset of those with anatomical compression are symptomatic, thus meeting criteria for MALS [5].

Common symptoms include postprandial pain, nausea, vomiting, and weight loss—often mistaken for functional gastro-intestinal (GI) disorders. The pain results from celiac plexus irritation as well as arterial narrowing, which explains why symptoms can occur even with minor stenosis [6, 7]. Compression worsens during expiration, making dynamic imaging vital.

Many patients undergo unnecessary GI evaluations before vascular causes are considered [7]. Doppler ultrasound, CT and MR angiography, digital subtraction angiography (DSA) are commonly used in combination to diagnose MALS. With improved non-invasive imaging, DSA is now mainly used when other tests are inconclusive or during planning of endovascular procedures [7]. Laparoscopic decompression, is described in current literature which involves cutting the median arcuate ligament and optionally performing celiac plexus neurolysis. We here describe a case of a 32-years-old lady with symptoms of MALS.

## Case presentation

A 32-year-old woman presented to our outpatient department with complaints of dull, cramping upper abdominal pain for 6 months, worsening after meals. The pain was associated with early satiety, nausea, and a weight loss of 9 kg during this period. There was no history of vomiting, melena, jaundice, or change in bowel habits. Her surgical and family history was unremarkable. On examination, she was of average built and poorly nourished with body mass index (BMI) of 15.4, with mild epigastric tenderness but no palpable mass or bruit. Routine laboratory investigations including complete blood count, liver and renal function tests were within normal limits.

**Figure 1:**
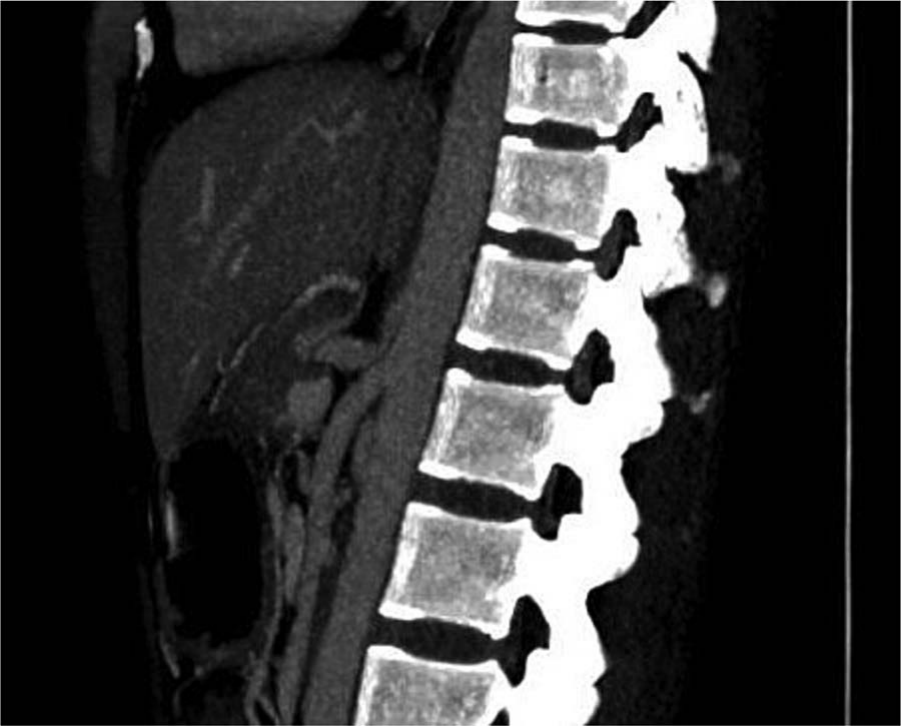
Showing marked focal narrowing at the origin of celiac trunk superior aspect by the arcuate ligament followed by post stenotic dilatation.

Initial scanning with abdominal ultrasound was inconclusive. Subsequently contrast-enhanced computed tomography (CECT) of the abdomen was done, which showed a marked focal narrowing at the origin of celiac trunk superior aspect by the arcuate ligament followed by post stenotic dilatation. No evidence of atherosclerosis seen (Fig. 1).

**Figure 2:**
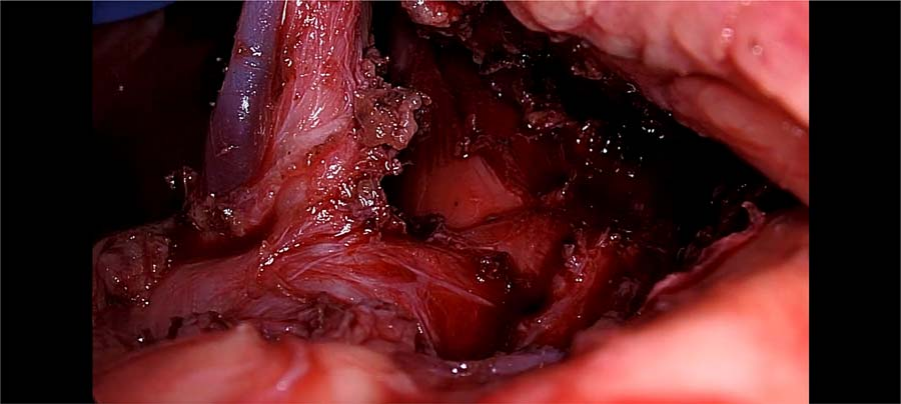
Showing intra-operative thick fibrous bands of median arcuate ligament crossing over the celiac trunk.

After multidisciplinary clinic-radiological discussion, the patient was counseled and consent was taken for Laparoscopic intervention. She underwent laparoscopic release of the median arcuate ligament. The intra-operative findings were thick fibrous bands of median arcuate ligament crossing over the celiac trunk, and indenting it antero-posteriorly at its origin from abdominal aorta. The fibrous band of meadian arcuate ligament were released from celiac trunk. The segment distal to compressed segment pulsations of celiac artery showed return of normal pulsations (Fig. 2). The post-operative course was uneventful. Her symptoms significantly improved by the second postoperative week, and she began regaining weight at 1-month follow-up.

## Discussion

MALS, or celiac artery compression syndrome, is a rare condition caused by external compression of the celiac artery by the median arcuate ligament. Despite being described in the 1960s, it remains difficult to diagnose due to vague symptoms and overlap with other abdominal conditions [8]. First described by Harjola in 1963 and further characterized by Dunbar *et al*. in 1965, MALS is defined by symptomatic vascular compression rather than anatomical findings alone [3, 8]. The median arcuate ligament is a fibrous arch anterior to the aortic hiatus, and in 10%–24% of people, it compresses a high-origin celiac artery [1].

The typical triad—postprandial epigastric pain, weight loss, and abdominal bruit—is suggestive but not definitive [1]. Our patient had postprandial pain, early satiety, nausea, and notable weight loss over 6 months, mimicking functional GI disorders and delaying diagnosis. MALS symptoms arise from both reduced blood flow and nerve compression at the celiac plexus, explaining the varied presentation and treatment responses.

Diagnosis depends on imaging, with CT angiography and Doppler ultrasound showing narrowing of the celiac artery and post-stenotic dilation, often worse during expiration [9]. While digital subtraction angiography (DSA) has been the gold standard, offering dynamic and respiratory phase evaluation [7], it is now used selectively due to its invasive nature. In our case, CECT with arterial phase confirmed celiac compression without signs of atherosclerosis.

Treatment is primarily surgical. Baccari *et al.* describes laparoscopic release of the ligament decompresses the artery and alleviates neurogenic pain [10]. Our patient experienced rapid symptom relief and nutritional improvement, consistent with recent reports. However, not all patients improve post-surgery. Persistent symptoms may stem from delayed diagnosis, lasting nerve damage, or coexisting GI disorders [5]. Thus, careful evaluation and multidisciplinary input are crucial to good outcomes. For residual stenosis, angioplasty or bypass may be added [11, 12]. Over 70% of patients experience symptom relief postoperatively [11].

Best outcomes are achieved when patient presents early with classic symptoms. Sultan *et al*. reported symptom relief in over 80% of patients treated with decompression and neurolysis [8]. Delayed diagnosis may reduce surgical success due to central sensitization [13]. In our case, her symptoms of post parandial pain, fear of food improved in second week of post-operative period and start regaining weight after 1 month of surgery. Her BMI raised from 15.4 to 18.3 after 3 months.

In some cases, angioplasty with or without stenting may help, though stents carry risks due to dynamic compression, including restenosis or fracture [14]. For neuropathic pain, image-guided celiac plexus block or sympathetic ablation may offer relief [5].

Persistent symptoms after laparoscopic median arcuate ligament release (MALR) require targeted evaluation. Post-operative imaging such as CT angiography or DSA is key to detect incomplete decompression, fibrotic stenosis, or rebound compression. If residual narrowing is found, revision surgery or vascular reconstruction like celiac artery reimplantation may be needed [6].

Functional GI disorders—like dyspepsia, Inflammatory bowel syndrome (IBS), or motility issues—should be ruled out. Gastroenterology referral for motility studies or prokinetic trials can guide treatment [15]. In chronic cases with weight loss, psychological evaluation, and support from pain specialists or behavioral therapists may be beneficial.

Given MALS’s rarity and varied presentation, prompt imaging and a multidisciplinary approach are essential. Long-term follow-up ensures symptom control and nutritional recovery.

## Conclusion

MALS, although rare, should be considered in the differential diagnosis of chronic postprandial abdominal pain and unexplained weight loss, especially in young adults. A high index of suspicion, supported by appropriate imaging, is crucial for timely diagnosis. Laparoscopic release of the median arcuate ligament offers a minimally invasive and effective treatment option, which restores quality of life, as demonstrated in our case. Continued follow-up is essential to monitor for symptom recurrence and to ensure long-term patient well-being.
